# Internal attention is the only retroactive mechanism for controlling precision in working memory

**DOI:** 10.3758/s13414-022-02628-7

**Published:** 2022-12-19

**Authors:** Fatih Serin, Eren Günseli

**Affiliations:** 1 MRC Cognition and Brain Sciences Unit, University of Cambridge, Cambridge, UK; 2 Department of Psychology, Sabanci University, Istanbul, Turkey

**Keywords:** Retro-cue, Selective attention, Reward, Cognitive control, Visual working memory

## Abstract

Recent research has suggested that humans can assert control over the precision of working memory (WM) items. However, the mechanisms that enable this control are unclear. While some studies suggest that internal attention improves precision, it may not be the only factor, as previous work also demonstrated that WM storage is disentangled from attention. To test whether there is a precision control mechanism beyond internal attention, we contrasted internal attention and precision requirements within the same trial in three experiments. In every trial, participants memorized two items briefly. Before the test, a retro-cue indicated which item would be tested first, thus should be attended. Importantly, we encouraged participants to store the unattended item with higher precision by testing it using more similar lure colors at the probe display. Accuracy was analyzed on a small proportion of trials where the target-lure similarity, hence the task difficulty, was equal for attended and unattended items. Experiments 2 and 3 controlled for output interference by the first test and involuntary precision boost by the retro-cue, respectively. In all experiments, the unattended item had lower accuracy than the attended item, suggesting that individuals were not able to remember it more precisely than the attended item. Thus, we conclude that there is no precision control mechanism beyond internal attention, highlighting the close relationship between attentional and qualitative prioritization within WM. We discuss the important implications of these findings for our understanding of the fundamentals of WM and WM-driven behaviors.

## Introduction

Visual working memory (VWM) is the mental workspace for the maintenance and manipulation of visual information. One considerable line of research regarding VWM is on the limited capacity of this system (Luck & Vogel, 2013). While earlier research focused on the number of items that can be maintained in VWM (Cowan, 2001; Luck & Vogel, 1997), recent work began to also investigate the precision of these items (Bays et al., 2009; Bays & Husain, 2008; Fougnie et al., 2016; Klyszejko et al., 2014; Zhang & Luck, 2008). These studies have shown that participants can voluntarily adjust the precision of VWM items according to task demands (Machizawa et al., 2012). Moreover, they have indicated that precision is higher for attended compared to unattended items (Bays et al., 2009; Gunseli et al., 2015; Klyszejko et al., 2014; van Moorselaar et al., 2015). Together, these findings suggest that attending to VWM items boosts their precision.

Although previous literature observed a close relationship between internal attention and precision, it is possible that precision is not a direct consequence of directed internal attention. There are two lines of argument to support this claim. First, manipulations of internal attention also encourage higher precision. For example, items that are cued are usually more likely to be tested, thus they benefit more from higher precision representations. Thus, participants in previous studies might have boosted memory representations via cognitive mechanisms such as working memory (WM) slots or resources (Bays & Husain, 2008; Zhang & Luck, 2008), which, in theory, can be allocated independently of internal attention. Second, several recent studies have indeed demonstrated a dissociation between storage and internal attention in WM (Günseli et al., 2019; Gunseli et al., 2015; Hakim et al., 2019; van Driel et al., 2017), suggesting that internal attention can be dissociated from maintenance. Taken together, whether precision is a direct consequence of internal attention or can be adjusted independently remains unknown. Given the importance of WM for other cognitive functions (Fukuda et al., 2010; Unsworth et al., 2014), mental health (Christopher & MacDonald, 2005; Fleming et al., 1997; Pan et al., 2011), and behavioral guidance (Olivers et al., 2011; Soto et al., 2008; Woodman & Chun, 2006), understanding the fundamentals of WM is essential.

To explore a mechanism of precision control in WM beyond internal attention, the present study attempted to manipulate precision independently of internal attention. Experiment 1 tried to accomplish this with task difficulty and test order. In every trial, participants had to keep two colors in mind, and a retro-cue indicated which color was going to be tested first, hence, manipulating internal attention (Ester et al., 2018; Griffin & Nobre, 2003; Günseli et al., 2019; Lepsien & Nobre, 2007). Importantly, we encouraged participants to remember the unattended color with higher precision by setting a higher similarity between the lure colors and the target color in the memory test for the uncued color (Machizawa et al., 2012). We further incentivized remembering the unattended color with higher precision by explicitly instructing individuals to do so, providing verbal reminders if their performance for the uncued item was worse than the cued item, and by providing reward for correct responses for the uncued item, as informed by unpublished work in our lab. The manipulation of precision (task difficulty) was applied simultaneously with and against the manipulation of internal attention (test order) to prevent the alternative explanation that the precision demand was fulfilled via internal attention. To control for the effects of perceptual and output interference caused by the first memory test, Experiment 2 reversed the order of the memory test in some trials. That is, in some trials, the item instructed to be stored with high precision was tested first. This way, we could evaluate the effects of precision manipulations in the absence of output interference by the first test. In Experiment 3, cued items were those that were tested second and required higher precision. By doing so, we evaluated whether retro-cue prevents participants from employing their VWM resources according to the task demands due to the automatic use of the retro-cue for prioritization in WM (Berryhill et al., 2012; Schmidt et al., 2002). Lastly, Experiment 4 provided a manipulation check for the task difficulty procedure used in Experiments 1, 2, and 3.

Comprehending the relationship between precision and attention in VWM can inform our understanding of the resources of WM. Although WM capacity is limited, what exactly is limited remains unclear. Most research states or implies that WM resources are either equivalent to or distributed via attention, suggesting that attention constitutes the limitation in WM (Awh & Jonides, 2001; Bays & Husain, 2008; Emrich et al., 2017; Kiyonaga & Egner, 2013; van den Berg et al., 2012). This predicts that the control of precision is a direct consequence of attention. On the other hand, some research argues that attention and WM resources do not completely overlap and that there are independent processes for controlling the storage of WM items beyond internal attention (Fougnie & Marois, 2006; Rerko & Oberauer, 2013). Given this dissociation between storage and attention, the precision of stored information may be controlled by a mechanism that does not include attention. Instead, storage-related WM resources may be responsible for adjusting precision hypothetically independently of the allocation of internal attention. Our study provides a venue to test these predictions and contribute to our understanding of the mnemonic units in WM.

The demarcation of precision and attention is not only important for our understanding of the mechanisms of WM storage, but also has implications for behavioral guidance by VWM. VWM has the capacity to bias visual attention such that the stimuli that match WM contents capture attention (Downing, 2000; Soto et al., 2005; Wolfe, 2007). However, there is an ongoing debate regarding the determining factor of guidance of external attention by a VWM item. While traditionally it has been suggested that internal attention dictates whether a VWM item will bias attention by assigning that item the status of an *attentional template* (Hollingworth & Hwang, 2013; Olivers et al., 2011), a recent theory challenged this template theory and argued that the precision of the items accounts for guidance without requiring the notion of a template status (Williams et al., 2022). What makes this a persisting debate is the aforementioned close relationship between internal attention and precision (Bays et al., 2009; Klyszejko et al., 2014). The present study creates a path to discover if this debate can be ultimately resolved by manipulating precision in opposition to internal attention.^1^

To summarize, through creating opposing internal attention and precision requirements, the present study attempted to explore whether precision is a direct outcome of internal attention or can be adjusted via a separate control mechanism. The findings highlight and contribute to our understanding of an overlooked gap in the nature of WM resources that carries implications for the debates surrounding behavioral guidance by VWM.

## Method

### Participants

All participants were Sabanci University students who received course credit in return for their participation. In Experiment 1, 13 (age range: 19–23 years, mean: 21.62 years; 11 female), in Experiment 2, 10 (age range: 19–24 years, mean: 20.8 years; six female), in Experiment 3, 16 (age range: 20–24 years, mean: 21.44 years; 14 female), and in in Experiment 4, 20 (age range: 18–26 years, mean: 21.2 years; 17 female) participants were recruited. None of the participants reported neuropsychological disorders or color blindness. Although exclusion criteria of 40% accuracy for the memory test (chance level = 33%) and 55% accuracy for the search task (chance level = 50%) were set, none of the participants were below these criteria. The experiments were approved by the Sabanci University Research Ethics Committee (SUREC) and were carried out according to the principles of the Declaration of Helsinki. Participants signed a consent form before the experiment.

### Stimuli

Experiments 1, 2, and 3 were created using the PsychoPy toolbox (Peirce et al., 2019) in order to carry out the experiment online through internet browsers. Therefore, there was a variance in the screen properties as people used their personal computers to perform the experiment. Experiment 4 was created with the Psychophysics Toolbox (Brainard, 1997; Pelli, 1997) on Matlab. Participants were instructed to remain seated 60 cm away from the screen. The background color was gray (hue saturation value - HSV: [0, 0, .5]). Filled colored circles (diameter 0.8°)^2^ were used as memory items. A fixation cross remained at the center of the screen throughout the trial. The fixation cross and all the texts (instructions and feedback) were black (HSV: [0, 0, 0]). On the memory display, two memory items were placed on the left and right sides of the fixation. A triangle (width 0.3°) was used as the retro-cue and shown slightly above the fixation. During the search task, eight lines were distributed evenly around the center. While the distractors were either horizontal or vertical, the target line was tilted 10°. During the memory test screen, three alternatives were located 0.8° above the fixation and were 0.5° apart. Memory item colors were picked randomly from among 360 different hues, all of which had the same saturation (.7) and value (.7) in the HSV model. The lure colors on the memory test screen had a hue difference of 28° on the easy, 38° on the medium, and 48° on the difficult condition.

## Experiment 1

### Procedure

**Fig. 1:**
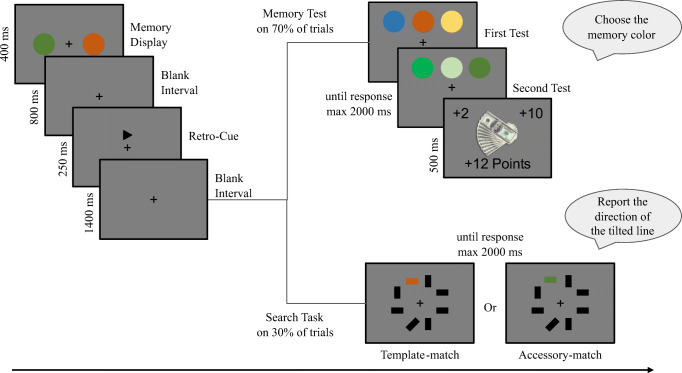
Example trial flow. *Note*. Depiction of a sample trial flow from Experiment 1. Two colored circles appeared on the memory display, and a retro-cue pointed to the location of one of the colors after a short retention interval to indicate the first test target. Following another retention interval, in 70% of the trials, the cued item was probed. Then, the second memory test appeared for the uncued color. In the remaining 30% of the trials, the search task was presented. After the response in either task, a feedback screen displayed accuracies. For memory trials, the feedback screen also included the earned points

Figure 1 depicts an example trial flow. Each trial started with the presentation of two random (controlled for a within-trial difference of at least 150° and between-trial difference of at least 45°) memory items for 400 ms. Then, a blank screen for a retention interval of 800 ms was shown. Following this, a retro-cue randomly pointed to one of the memory item positions, indicating the first item to be tested. After another retention interval of 1,400 ms, the memory test was probed in 70% of the total trials, and the search task was probed in the remaining 30%.

During the memory test, participants were first asked to choose the cued color among three alternatives. After the response, participants were asked to pick the uncued color among three alternatives. They used “J,” “K,” or “L” keys to pick the left, middle, or right option, respectively. Critically, participants were instructed that the first test would be easy, and the second test would be difficult due to the similarity of the lure colors to the correct color on the test display. However, in 25% of these memory trials, both tests were set to medium difficulty to have comparison trials where both the first and the second tests can be evaluated without the test difficulty confound. For both memory tests and the search task, participants had 2,000 ms to respond. After their response to the second memory test or the search task, they received feedback for their accuracy. Additionally, participants were also instructed that memory tests would be rewarded with points. The first test would be rewarded 2 points while the second test would be rewarded 10 points to assist the difficulty manipulation and encourage participants to remember the uncued item with higher precision. The points received during a trial were shown on the feedback screen. If participants were able to respond to both the first and the second test accurately, the feedback screen displayed an image of US banknotes for additional motivation, though no actual payment was made and participants were aware of this. The search task was not rewarded. To further support the difficulty and reward manipulation, after every five medium difficulty trials, participants received a warning on the feedback screen if they performed worse for the uncued item than the cued item based on these last five medium difficulty trials. The feedback screen lasted for 300 ms unless participants received this warning or failed to respond within the time limit, in which case the time window was extended to 1,000 ms. Including the feedback, the inter-trial interval lasted 1,500 ms.

During the search task, participants were asked to find the tilted line among horizontal and vertical distractors and report whether the tilt is toward left or right by pressing “J” or “L” for left tilt or right tilt, respectively. Critically, in every search trial, one of the distractors was colored. In half of these search trials, the colored line matched the cued item, template-match condition, and in the other half, the colored line matched the uncued item, accessory-match condition. These trials would allow us to observe attentional guidance by different representations in WM via slower reaction times (RTs) due to the distractor matching memory contents.

Participants performed 12 practice trials prior to the main experiment. Participants had to repeat the practice phase if they could not perform above chance level for either the memory or the search tasks (Experiment 1: minimum repetitions = 1, maximum repetitions = 7, median = 1.5; Experiment 2: min = 1, max = 3, median = 1.5; Experiment 3: min: 1, max, 7, median = 2). The main experiment had 560 trials in total, 392 of which were memory trials and 168 of which were search task trials. Out of 392 memory trials, there were 98 medium-difficulty trials.

### Results

**Fig. 2:**
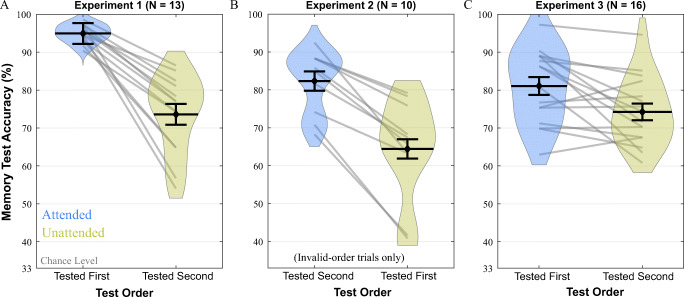
Memory test accuracy results. *Note.* Violin plots for memory test accuracies in medium difficulty trials in Experiments 1 (**A**), 2 (**B**), and 3 (**C**). Error bars represent one standard error of the within-subject attended versus unattended difference

The accuracies for medium difficulty first and second test results are shown in Fig. 2A. Accuracies for the memory tests were calculated as the percentage of correct answers in medium difficulty trials (this applies to the following experiments as well). A paired-samples t-test was conducted to compare first and second memory test performances. Accuracy for the first test (*M* = 94.94, *SD* = 2.4) was higher than the second test (*M* = 73.6, *SD* = 10.4). The difference, 21.35 (95% CI [15.33, 27.36]; Cohen’s *d* = 2.14), was significant according to the paired-samples t-test, *t*(12) = 7.73, *p* < .001. A Bayesian paired-samples t-test produced BF_10_ = 3812.305.

### Discussion

Experiment 1 demonstrated that participants performed better for the cued item (first memory test) than the uncued item (second memory test) in medium trials despite the task requirements and instructions demanding the opposite. The results might indicate that precision is the direct consequence of internal attention since attending to one item made it impossible to maintain another item with higher precision. However, the perceptual and output interference from the first memory test potentially disrupts the precision of the uncued item, creating an alternative explanation for the lower precision in the second memory test. To assess the item precisions without these interferences, we designed Experiment 2, which reverses the order of memory tests in a minority of the trials.

## Experiment 2

### Procedure

To free the precision assessment in the second memory test from the perceptual and output interference from the first test, we made the retro-cue probabilistic in Experiment 2. Experiment 2 had three main differences compared to Experiment 1. First, to create trials where the assessment of the cued item did not interfere with the assessment of the uncued item, retro-cue validity was reduced from 100% to 75%. In 25% of the trials, participants were given the memory tests in reverse order, uncued item first and cued item second. As in Experiment 1, to preserve statistical power, these reverse-order trials were also set as the medium difficulty trials, as they were the trials analyzed to compare cued and uncued item precisions. Second, because retro-cue was not deterministic anymore, during the test screens, a white circle indicated the item that was currently being tested by showing up at the position where that item appeared during the memory display. Lastly, to increase the statistical power and prevent fatigue, the search task was not included.

### Results

Accuracies are plotted in Fig. 2B. Accuracy for the second tested cued item memory test (*M* = 82.33, *SD* = 8.34) was higher than the first tested uncued item memory test (*M* = 64.42, *SD* = 13.52). The difference, 17.92 (95% CI [12.16, 23.68]; Cohen’s *d* = 2.23), was significant, *t*(9) = 7.04, *p* < .001. Furthermore, BF_10_ = 436.724 was acquired according to a Bayesian paired-samples t-test.

### Discussion

Experiment 2 showed that the second tested cued item, which was subject to perceptual and output interference, had better precision compared to the first tested uncued item, which was free from interference. This finding is in parallel with Experiment 1 and suggests that internal attention is the mechanism that controls precision, because while subject to interference, the attended item had higher precision compared to the unattended item that was tested without interference. Therefore, Experiment 2 suggests that perceptual and output interference does not provide an alternative explanation for the Experiment 1 results that displayed lower precision for the unattended but incentivized item compared to the attended item. Besides interference, retro-cue manipulation might also provide an alternative explanation. It was shown that retro-cue effects on precision are, at least partially, automatic (Berryhill et al., 2012; Schmidt et al., 2002). Based on this, retro-cue effects might be too strong on WM such that it prevents maintenance of the unattended item. Thus, Experiment 3 reversed the function of the retro-cue to control for the automatic effects of the retro-cue.

To evaluate the effects of not experiencing perceptual and output interference costs for uncued items, we compared accuracy in this condition to that in Experiment 1. There was no significant difference even though numerically the accuracy was lower in Experiment 2 where interference was absent. This counterintuitive trend could reflect either individual differences across experiments or performing the reverse order test infrequently (25% of trials). In other words, in Experiment 1, participants knew which item they would be tested for, whereas in Experiment 2, the test order was uncertain. The lack of certainty in test order could have been detrimental for performance.

## Experiment 3

### Procedure

Experiment 3 was identical to Experiment 1 except for only one aspect. In this experiment, the function of the retro-cue was reversed such that it pointed to the item that was going to be tested second and was going to require high precision, rather than pointing to the first tested (attended) item. Participants were informed about this prior to the experiment.

### Results

The accuracies are shown in Fig. 2C. The average accuracy was higher for the attended item (*M* = 81.08, *SD* = 9.65) compared to the unattended item (*M* = 74.24, *SD* = 9.051), *t*(15) = 2.88, *p* = .011, (95% CI of the difference = [1.77, 11.9]; Cohen’s *d* = 0.72). Additionally, a Bayesian paired-samples t-test yielded BF_10_ = 4.876.

### Discussion

Experiment 3 reversed the function of the retro-cue to control for the automatic retro-cue effects that might have biased precision control mechanisms in favor of the cued item. The results replicated the findings in Experiment 1 and 2, as the accuracy was higher for the attended (this time uncued) item. Thus, Experiment 3 suggests that storing an unattended item with higher precision than the attended item is not possible, despite task demands, reward, and feedback encouraging it to be stored more precisely.

However, although we see that the results are, on average, in line with the previous two experiments, we do not see the exact participant-level consistency in the data. While all participants showed the same pattern in Experiments 1 and 2, in Experiment 3, four out of 16 participants did not display the average pattern that all the experiments showed. This deviation could be due to misunderstood instructions that led participants to think they should direct their internal attention to the second tested item pointed by the cue rather than the first tested item. Alternatively, as previously suggested, retro-cues may create a strong automatic prioritization bias (Berryhill et al., 2012; Schmidt et al., 2002) that might make it harder to allocate attention to the item opposite to it, at least for some individuals. Despite possibly reflecting a weaker effect – which we did not confirm via a between-subjects comparison due to the low number of participants in each experiment – Experiment 3 replicated the pattern of the previous two experiments: accuracy was higher for the attended item. Thus, we conclude that precision cannot be adjusted via a mechanism beyond internal attention.

## Experiment 4

### Procedure

In this experiment, different from the previous three experiments, only one of the items was tested in each trial and the cue showed which item would be tested with a difficult test if it was tested. The item to be tested and the item that the cue pointed to was picked randomly in every trial. Participants were instructed that the cue informs which item will be tested with a difficult test but not *if* it will be tested. The test difficulty manipulation was the same as the previous experiments. If the cued item was tested, the alternatives were more similar to the item and if the uncued item was tested, the alternatives were less similar. Again, on 25% of these trials, intermediate difficulty was used to make a fair comparison between the cued and the uncued item.

### Results

**Fig. 3:**
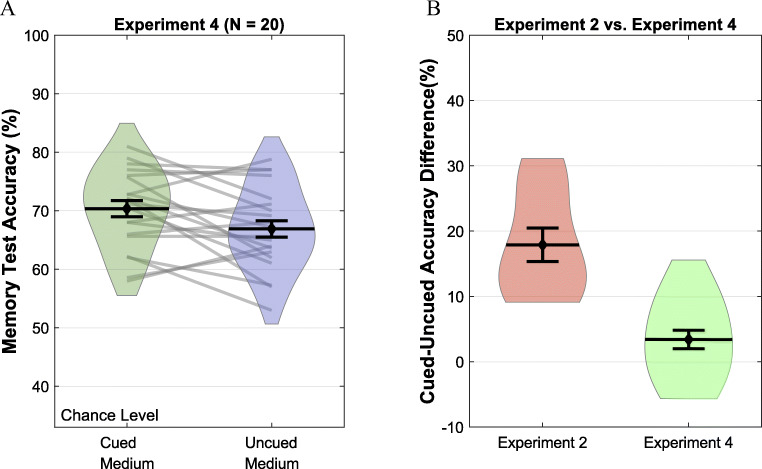
Memory test accuracy results. *Note.* Violin plots for memory test accuracies in medium difficulty trials in Experiments 4 (**A**). Error bars represent one standard error normalized for within-subjects variance. Violin plots for the comparison of the accuracy differences in Experiments 2 and 4 (**B**). Error bars represent one standard error of between-subjects variance

Accuracy (Fig. 3A) was higher for the cued item (*M* = 66.89, *SD* = 7.14) compared to the uncued item (*M* = 70.34, *SD* = 6.68), *t*(19) = 2.43, *p* = 0.0253, (95% CI of the difference = [0.47, 6.41]; Cohen’s *d* = 0.54). A Bayesian paired-samples t-test provided BF_10_ = 2.39.

### Discussion

**Fig. 4:**
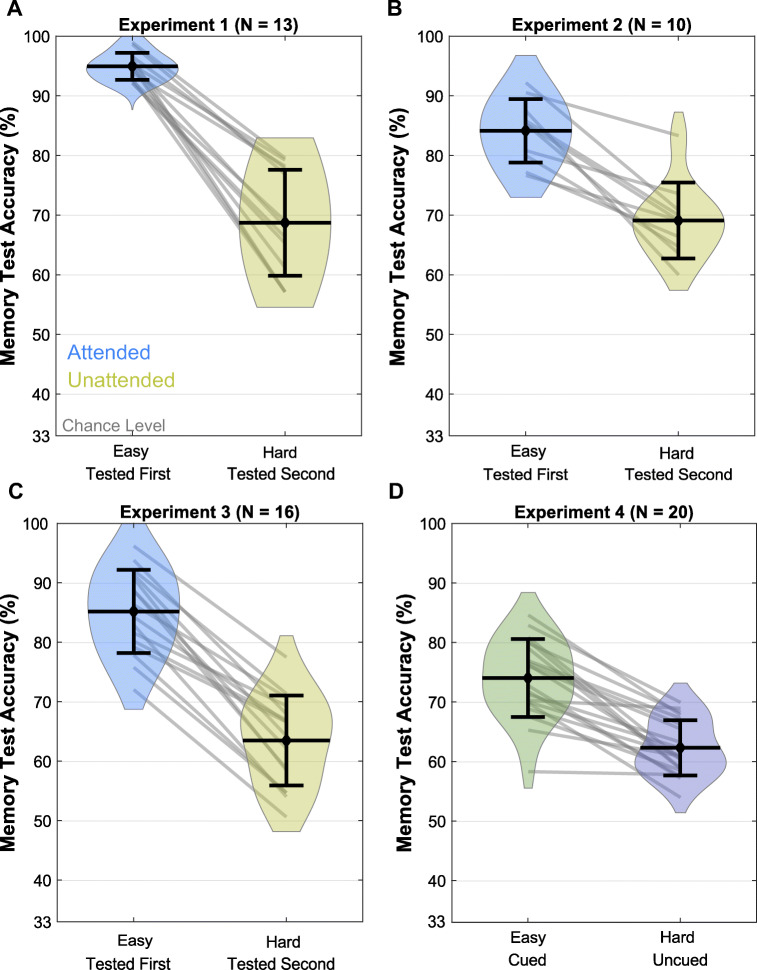
Memory test accuracies for the easy and hard difficulty trials. *Note.* Violin plots for memory test accuracies in easy and hard difficulty trials in Experiments 1 (**A**), 2 (**B**), 3 (**C**), and 4 (**D**). Error bars represent one standard error of between-subjects variance

Experiment 4 was carried out to ensure that the lure-target similarity manipulation was able to encourage participants to adjust the precision of the items in WM. Participants were more accurate for the item that was going to be tested with a difficult test compared to the item that was uncued. Based on this result, we conclude that this manipulation is indeed able to encourage participants to remember the item that will be tested in a higher target-lure similarity with higher precision. In line with this conclusion, the easy and hard trials of all four experiments also demonstrate that higher target-lure similarity results in lower accuracy for the items. These trials are plotted in Fig. 4.

### Aggregate analysis

To increase our power, we combined data from Experiments 1, 2, and 3 and ran the same tests that were run for each experiment individually. For these 39 participants, The average accuracy was significantly higher for the attended item (*M* = 86.02, *SD* = 9.81) compared to the unattended item (*M* = 71.51, *SD* = 11.3), *t*(38) = 8.07, *p* < .001.; 95% CI of the difference = [10.87, 18.16]; Cohen’s *d* = 1.29). The Bayesian paired-samples t-test provided extremely strong evidence in favor of the hypothesis that accuracy was higher for the attended item (BF_10_ > 12300000). Moreover, 35 out of 39 participants had higher accuracy for the attended item. Thus, we claim that our effect is robust and reliable.

We ran a post hoc power analysis to estimate the acquired statistical power of this aggregate analysis. We used the G*Power software (Faul et al., 2009) with α = .02, two-tailed, sample size 39, and we chose the smallest effect size we acquired across experiments (Experiment 3: Cohen’s *d* = 0.72). The statistical power of this aggregate analysis was 97.8%, which suggests that our study, across three experiments, had a high power to test our hypothesis.

### Is there a weak precision control mechanism that partially counteracts the effects of internal attention in experiments 1–3?

Even though the results of Experiments 1, 2, and 3 point to internal attention as the only precision control mechanism, there is a possibility that task difficulty does influence precision independently of internal attention. Our results can also be explained by two distinct sources of precision boost, internal attention and a separate precision control mechanism, if they can act simultaneously and the effect of internal attention is larger than the separate control mechanism. To evaluate this possibility, we compared the accuracy difference between the cued and the uncued items across Experiments 2 and 4 (Fig. 3B). Experiment 2 was chosen as the comparison experiment because it also involves an uncertainty in the test order. If the task difficulty can control precision independently of internal attention, Experiment 2 should have a smaller cued and uncued item difference compared to Experiment 4, as Experiment 2 has two opposing mechanisms counteracting each other. In contrast to this prediction, the cued and uncued item difference in Experiment 2 (*M* = 17.92, *SD* = 8.05) was significantly *larger* than the cued and uncued difference in Experiment 4 (*M* = 3.44, *SD* = 6.34) according to an independent-samples t-test, *t*(28) = 2.43 , *p* < .001.; 95% CI of the difference = [8.98, 19.98]; Cohen’s *d* = 2.09). A Bayesian independent-samples t-test produced BF_10_ = 1517.43.

Rather than having a smaller difference, Experiment 2 had a substantially higher difference compared to Experiment 4. Therefore, this provides evidence that precision control is established via internal attention and not controlled by an independent mechanism. The cued-uncued difference in Experiment 4 being smaller than in Experiment 2 could be due to difficulty not being as critical as test order for participants. Consequently, participants could have deployed less attention to the cued item when the cue informs only about the difficulty and not the order.

## General discussion

We investigated the existence of a precision control mechanism beyond internal attention in three experiments. The experiments aimed to contrast – within the same trial – internal attention, directed via a retro-cue, and precision demands adjusted via target-lure similarity in a three-alternative forced-choice task. In three experiments, we found that the internally attended item had higher precision despite encouraging participants to store the unattended item with higher precision via task demands (i.e., the higher similarity between the memory and lure colors), instructions, feedback, and reward. This result held despite controlling for the perceptual and output interference on the unattended item (Experiment 2) and automatic retro-cue effects on the attended item (Experiment 3). When not counteracted by attention, expectation of higher task-lure similarity did result in higher precision, suggesting that our task difficulty manipulation was effective as long as it did not have to counteract internal attention (Experiment 4). Our results reflect the dominance of internal attention in controlling the precisions of items in WM and suggest that internal attention is the sole control mechanism of WM precision.

In this study, we tested a voluntary control mechanism of precision beyond internal attention. The lack of a voluntary control mechanism does not preclude the possibility of other, indirect factors that affect WM precision. However, we argue that internal attention may account for most of the other factors claimed to affect WM precision. First, while it may be possible that physical properties of stimuli and statistical regularities that signal importance of given items or locations (e.g., being often tested or rewarded) can determine which item will be encoded or remembered with higher precision, previous studies have found that these factors typically determine the probability of remembering these items and not their precision (Ravizza et al., 2021; Umemoto et al., 2010). Thus, implicit importance signals may be specific to all-or-none storage probabilities and not the quality of the stored items. Second, factors such as memory load (Zhang & Luck, 2008), delay duration (Pertzov et al., 2017), and perceptual interference (Makovski & Pertzov, 2015; van Moorselaar et al., 2015), which have been found to influence precision, may do so through their influence on the distribution of internal attention. For example, less attention can be devoted to a given item with increasing memory load, when attention is directed to distracting stimuli, and to other external stimuli and internal thoughts with increasing retention intervals. That is, attention can be the moderator of all the other factors that are known to influence precision except factors that determine the availability of sensory input such as masking during encoding. Future work is needed to isolate the role of attention from these other factors in determining the precision of WM representations. In line with this, our claim does not argue that the previous manipulations of task difficulty (Machizawa et al., 2012) to influence precision were ineffective. Rather we argue that the difficulty requirement may have influenced precision via internal attention. Taken together, the scope of our claims in this study covers top-down and retrospective mechanisms for controlling precision in WM.

Although we claim that internal attention is the only predictor of precision in WM, it is important to note that precision can be higher for currently unattended but previously well-learned information. Previous studies have shown that repeatedly remembering the same information results in its handoff to long-term memory (Carlisle & Woodman, 2011; Gunseli et al., 2016; Gunseli, Meeter, et al., 2014a; Gunseli, Olivers, et al., 2014b; Reinhart et al., 2014). However, the precision of information increases despite this handoff being associated with less attention (Reinhart & Woodman, 2014; Serin & Günseli, in prep; van Moorselaar et al., 2016). In line with this, while we propose that internal attention is the main predictor of precision in WM across items of similar familiarity, previously represented information can be stored with high precision despite receiving little internal attention in a given instance. Arguably, this is due to having devoted sufficient internal attention to this information in the past. To better isolate the unique role of each, future studies will need to explore the distinct contributions of internal attention distributed in a given moment and the cumulative internal attention devoted in the past for determining WM precision.

Our conclusion that internal attention is the sole control mechanism of precision in WM is in line with the neural underpinnings of attention. Attention has been shown to be associated with regulatory feedback from higher-order regions to early sensory areas (Buschman & Miller, 2007; Martin et al., 2019; Moore & Armstrong, 2003). Such regulatory activity may determine how precisely each item will be represented by affecting the neural characteristics such as the extent to which neural populations represent information (Ester et al., 2016), the neural gain or tuning (Itthipuripat et al., 2014; Kanwisher & Wojciulik, 2000; Scolari et al., 2012; Sprague et al., 2015), and connectivity across regions (Al-Aidroos et al., 2012; Parks & Madden, 2013; Saproo & Serences, 2014). Although some of these attentional modulatory effects have been found in the perceptual domain, given the similarities of attention directed in the external world and internally in WM (Griffin & Nobre, 2003), it is likely that similar neuromodulatory effects of attention apply within WM.

Our interpretation of the results is based on intense testing of the hypothesis that precision and internal attention in WM are separable. It might be the case that there is a mechanism that controls precision in WM beyond internal attention, but it is, at least with our manipulations, unable to surpass the effects of internal attention. In other words, our design and criteria may not have been sensitive enough to observe this other mechanism. In line with this argument, recent work demonstrated that neural measures of WM activity and internal attention do not completely overlap (Günseli et al., 2019; Gunseli et al., 2015; Hakim et al., 2019; van Driel et al., 2017). Thus, there might be a WM control mechanism that operates independently of internal attention. Nevertheless, based on higher memory performance for the attended item, we can conclude that attention is the strongest determinant of precision.

Our findings inform the recent debate regarding what enables WM items to guide external attention. The traditional view held that internal attention creates an attentional template, and this template enables guidance by WM (Carlisle et al., 2011; Hollingworth & Hwang, 2013; Olivers et al., 2006, 2011; van Moorselaar et al., 2014). But a recent work proposed that precision of items enable them to guide attention and that the template concept is redundant (Williams et al., 2022). Considering our findings, it is possible that the debate stems from the inseparability of internal attention and precision in WM. If internal attention and precision are integral in WM, as our findings suggest, this would explain why there is a debate between internal attention and precision in WM-guided attention literature.

Recent work has questioned the dissociation between accessibility and precision of representations (Schurgin et al., 2020). The study has argued that a model with a single memory strength parameter can explain more variance of error in a continuous report task. Moreover, in the present study, we did not use a continuous report task to assess precision by fitting the distribution of errors in a mixture model (Zhang & Luck, 2008). Instead, we used accuracy in a three-alternative forced-choice task to draw conclusions regarding precision. Consequently, a higher probability of storage, instead of higher precision may have accounted for the higher accuracy for attended items. While we suggest that our conclusions are indirectly supported by the neurological and behavioral evidence that favors the distinction between accessibility and precision (Bays et al., 2009; Berens et al., 2020; Fan & Turk-Browne, 2013; Richter et al., 2016), and evidence that shows target-lure similarity affects the quality of items (Kim & Yassa, 2013; Motley & Kirwan, 2012), it is possible that the claimed effects of internal attention on precision may instead reflect the effects of attention on other aspects of memories. However, we believe that our findings are informative for “precisionless” models of WM. If higher accuracy in our task reflects higher memory strength or likelihood of storage, our results would suggest that these memory aspects are determined by internal attention even when they are contrasted by task demands, instructions, and reward. Thus, regardless of the assumptions regarding the existence of precision, our results highlight the close relationship between internal attention and WM storage.

Although our study dovetails with a range of studies that demonstrate the role of attention for storage in WM, we do not suggest that attention should be equated to storage in WM. Recently, Günseli et al. (2019) have shown that directing attention away from a WM item does not immediately result in the loss of this item. Specifically, by using contralateral alpha suppression and contralateral delay activity as indices of spatial attention and storage of items in WM (Hakim et al., 2019; Machizawa et al., 2012), Günseli et al. (2019) showed that an item’s probability of test determines whether it will be unattended and discarded from WM (low probability of being tested), or unattended but will be retained (relatively higher probability of being tested). Thus, we propose that attention is beneficial for the storage of information in WM, but it is not necessary for keeping information available, at least within the range of a few hundred milliseconds.

In summary, this study searched for a precision control mechanism beyond internal attention. In three experiments, despite encouraging the storage of an unattended item with higher precision than the attended one, the attended item was remembered better. Thus, we conclude that internal attention is the only control mechanism of WM precision. This finding contributes to the fundamentals of WM representations and the relationship between attention and WM.

## Data Availability

The experiments were not preregistered. The data and analysis scripts are accessible via the Open Science Framework at: https://osf.io/7bfqz/?view_only=84d81a3d1b4c4cab96250de7ed7e873a.

## Footnotes

1 In line with this, the second goal of the present study was to investigate the unique role of internal attention in guiding attention beyond its precision. To do so, our experiments also included a search task that contained memory-matching distractors. However, in follow-up experiments, we found that our search task did not reveal the well-established WM-driven involuntary attentional guidance even when there was only one item in WM. This was most likely due to the stimuli used in the search display being open instead of full circles (Olivers, 2009). Thus, our study was not fit to assess external attentional guidance. As a result, we will not discuss the results of the visual search task.

2 Due to lack of control over the visual angles during these online experiments (Experiments 1, 2, and 3), approximate visual angles are provided in reference to a typical 15.6-in. screen laptop with 1,920 × 1,080 resolution.
